# Detection of remaining *Plasmodium* DNA and gametocytes during follow up after curative malaria treatment among returned travellers in Norway

**DOI:** 10.1186/s12936-020-03367-6

**Published:** 2020-08-19

**Authors:** Christel Gill Haanshuus, Kristine Mørch

**Affiliations:** 1 Norwegian National Advisory Unit On Tropical Infectious Diseases, Unit for Infectious Diseases, Department of Medicine, Haukeland University Hospital, Bergen, Norway; 2 Department of Clinical Science, University of Bergen, Bergen, Norway

**Keywords:** Malaria, *Plasmodium falciparum*, *Plasmodium vivax*, Gametocyte, PCR, RT-PCR, mRNA, DNA, Travellers, Post-treatment

## Abstract

**Background:**

PCR can be positive weeks after effective malaria treatment, potentially leading to over diagnose of recrudescence and re-infections. The DNA detected by PCR post-treatment might stem from residuals of destroyed asexual parasites, or from live gametocytes. The objective of this clinical observational study was to describe the presence of positive PCR for *Plasmodium falciparum* and *Plasmodium vivax* in follow-up samples post-treatment from returned travellers, and the proportion of positive PCR due to gametocytes.

**Methods:**

Whole blood was collected during hospitalization and outpatient routine follow-up from 13 patients with imported malaria. DNA was extracted applying QIAamp DNA Blood Mini Kit, while mRNA was collected and extracted applying PAXgene Blood RNA Tubes and Kit. All DNA samples (N = 25) were analysed with a genus-specific *cytb* real-time SYBR PCR, and *P. falciparum* DNA samples (N = 22) were also analysed with a falciparum-specific *var*ATS real-time TaqMan PCR. All the mRNA samples (N = 18) were analysed with both a genus-specific 18S rRNA RT-PCR and a gametocyte-specific *Pfs*25 (*P. falciparum*)/*Pvs*25 (*P. vivax*) RT-PCR.

**Results:**

Latest samples were collected at day 1 (n = 2) and from day 11–54 (n = 11) after treatment. Genus DNA *cytb* PCR was positive up to 49 days after effective treatment, and 18S rRNA transcripts from active *P. falciparum* parasites were detectable for at least 11 days. Gametocyte-specific mRNA was detected at latest only two days after treatment. Among six patients with late positive PCR for *P. falciparum*, four had high parasitaemia at admittance (6–30%), while two had parasitaemia < 2%. Late detection of *P. vivax* was not found by any of the PCR methods.

**Conclusions:**

DNA-based PCR can be positive up to at least seven weeks after curative malaria treatment, potentially leading to over-diagnose of recrudescence and re-infections. Based on the observations in this study, it is unclear if the DNA origins from residuals of destroyed parasites or live gametocytes, warranting further investigations.

## Background

Polymerase chain reaction (PCR) detecting *Plasmodium* DNA is the most sensitive method for diagnosing malaria, and used as reference method for routine diagnostics and in epidemiological surveys. However, PCR can be positive weeks after effective malaria treatment, potentially leading to over-diagnose of recrudescence and re-infections [1]. Residuals of destroyed asexual parasites, and live or destroyed gametocytes, are possible sources of post-treatment *Plasmodium* DNA in patients cured of their malaria infection. Gametocytes are sexual forms of malaria parasites responsible for transmission between humans after completion of their cycle in mosquitoes. They cause no clinical symptoms in the human host. In *Plasmodium falciparum,* immature gametocytes sequester in internal host organs, particularly in the bone marrow, and undergo five morphological development stages in the course of 7–12 days, hence only mature gametocytes circulate in the blood until they die of age [2, 3]. It has been estimated from mathematical modelling that *P. falciparum* gametocyte carriage may persist for up to 55 days after treatment [4]. Primaquine (PQ) is the only drug effective against mature gametocytes, and is added to artemisinins in routine treatment of *P. falciparum* in many malaria-endemic areas to prevent transmission [5, 6]. Artemisinin also has some effect on gametocyte carriage duration, through the effect on immature gametocytes and rapid killing of asexual parasites [5]. Less is known about *Plasmodium vivax* gametocytes. It seems that immature *P. vivax* gametocytes sequester mainly in the bone marrow, similar to *P. falciparum* [7]. However they mature much faster than *P. falciparum* gametocytes, they are commonly present before symptoms and before parasite detection by microscopy, and live only for up to three days [2, 8].

Several malaria PCR methods have been introduced applying different amplification targets on the parasite genome. The real-time PCR targeting *var* gene acidic terminal sequence (*var*ATS) on the chromosomal genome, and *cytochrome b* (*cytb*) on the mitochondrial genome, are among the most sensitive ones [9]. The *var*ATS gene exists in 59 copies for each parasite nucleus [10], while the number of copies of the *cytb* gene varies depending on parasite stage; ring-stage parasites have about 20 copies, and gametocytes about 160 copies [11, 12]. Gametocyte-specific mRNA transcripts can be detected by reverse transcript (RT)-PCR methods [13, 14].

The objective of this clinical observational study was to describe the presence of positive PCR for *P. falciparum* and *P. vivax* in follow-up samples post-treatment from returned travellers, and the proportion of positive PCR due to gametocytes, applying PCR methods detecting *Plasmodium* DNA and gametocyte-specific mRNA.

## Methods

### Patient material

Blood samples from adult patients, diagnosed with malaria by microscopy and/or rapid diagnostic test at Haukeland University Hospital, Bergen, were collected in the period 2013–2015. Blood samples were collected during hospitalization and at routine follow-up in the outpatient clinic at 11–54 days after treatment. Clinical information and results from routine microscopy was collected retrospectively from patient records.

## PCR methods

DNA was extracted from EDTA whole blood applying QIAamp DNA Blood Mini Kit (Qiagen, Hilden, Germany), while mRNA was collected and extracted applying PAXgene Blood RNA Tubes and Kit (PreAnalytiX, Hombrechtikon, Switzerland), according to the manufacturer’s instructions.

All the DNA samples (N = 25) were analysed with a genus-specific *cytb* real-time SYBR PCR, and the *P. falciparum* DNA samples (N = 22) were also analysed with a *falciparum*-specific *var*ATS real-time TaqMan PCR, following previously published methods [9, 10]. All the mRNA samples (N = 18) were analysed both with a genus-specific 18S rRNA RT-PCR (detecting mRNA transcripts produced by all parasite stages), and a gametocyte-specific *Pfs*25 (*P. falciparum*)/*Pvs*25 (*P. vivax*) RT-PCR detecting female gametocytes, following previously published methods [14]. In addition, quantitative analysis was performed for the *cytb* real-time PCR and *Pfs*25/*Pvs*25 RT-PCR runs by applying tenfold dilution series of customized plasmids designed with EcoRI linearized q-PCR template and a pUCminusMCS vector backbone (OriGene Technologies, Rockville, MD, USA). All samples were run in triplicates, and a positive result was defined as at least two out of three detections.

## Results

The PCR results applying DNA and mRNA template, including mean C_t_ values as well as quantitative numbers, are presented in Table 1. Genus DNA *cytb* PCR was positive up to 49 days after effective treatment, and 18S rRNA transcripts from active *P. falciparum* parasites were detectable for at least 11 days. Microscopy and/or PCR detected gametocytes in 67% (8/12) patients, but gametocyte-specific mRNA was detected at latest only two days after treatment. Late detection of *P. vivax* was not found by any of the PCR methods.

**Table 1:** *Plasmodium* DNA and gametocyte-specific mRNA detections among malaria patients after treatment (N = 13)

Patient	Species	No days since treatment	Microscopy (parasite %)	Genus DNA *Cytb *PCR (C_t_)	No copies/rxn	*P.f *DNA *Var*ATS PCR (C_t_)	Genus 18S mRNA PCR (C_t_)	Pfs25/Pvs25 mRNA PCR (C_t_)	No. copies/rxn
No. 1	*P.f* + *P.m*	0	Pos (2)	Pos (24)	1.1 × 10^3^	Pos (26)			
		1	Pos (1)	Pos (25)	4.3 × 10^2^	Pos (28)	Pos (17)	Pos (29)	3.2 × 10^3^
No. 2	*P.f*	0	Pos (4)	Pos (18)	5.7 × 10^5^	Pos (19)			
		1	Pos (4)	Pos (21)	7.0 × 10^5^	Pos (22)	Pos (12)	Pos (34)	95
No. 3	*P.f*	0	Pos (12)						
		1	Neg	Pos (22)	3.7 × 10^4^	Pos (26)			
		2	Neg	Pos (23)	1.5 × 10^4^	Pos (29)			
		11	Neg	Pos (30)	200	Pos (35)	Pos (25)	Neg	
No. 4	*P.f*	0	Pos (< 1)*						
		31	Neg	Neg		Neg	Neg		
No. 5	*P.f*	0	Pos (< 1)						
		32	Neg	Pos (37)	2	Neg	Neg		
No. 6	*P.f*	0	Pos (2)	Pos (19)	3.9 × 10^5^	Pos (20)			
		1	Pos (< 0.5)	Pos (24)	7.7 × 10^3^	Pos (27)	Pos (17)	Pos (31)	420
		37	Neg	Pos (39)	1	Neg	Neg		
No. 7	*P.f*	0	Pos (10)	Pos (15)	4.0 × 10^6^	Pos (16)			
		4	Neg	Pos (25)	6.3 × 10^3^	Pos (29)	Pos (23)	Neg	
		11	Neg	Pos (29)	400	Pos (35)	Pos (24)	Neg	
		39	Neg	Neg		Neg	Neg	Neg	
No. 8	*P.f*	0	Pos (30)	Pos (16)	1.8 × 10^6^	Pos (18)			
		1	Pos (6)	Pos (16)	3.0 × 10^6^	Pos (17)	Pos (7)	Pos (29)	2.2 × 10^3^
		45	Neg	Pos (34)	16	Neg	Neg	Neg	
No. 9	*P.f*	0	Pos (6)*						
		49	Neg	Pos (34)	11	Pos (36)	Neg	Neg	
No.10	*P.v*	0	Pos (0.5)						
		2	Pos*				Pos (7)	Pos (17)	9.6 × 10^6^
No. 11	*P.v*	0	Pos (0.5)^a^						
		6	Neg	Pos (39)	1		Neg	Neg	
No. 12	*P.v*	0	Pos	Pos (22)	4.8 × 10^4^				
		23	Neg	Neg			Neg	Neg	
No. 13	*P.v*	0	Pos						
		54	Neg	Neg		Neg	Neg	Neg	

*rxn* reaction

^a^Gametocytes seen by microscopy

All patients were asymptomatic and had negative microscopy at outpatient follow-up visits. The association between positive PCR, microscopy findings and clinical characteristics is presented in Table 2. Six patients with *P. falciparum* were treated with artesunate intravenously, followed by a full oral course of artemether-lumefantrine (AL) or atovaquone-proguanil (AP), while two received only AL and one only AP. All *P. vivax* patients, and no *P. falciparum* patients, received primaquine. Among the six patients with late *P. falciparum* positive PCR (day 11–49), four had high parasitaemia (6–30%) while two had parasitaemia < 2% at admittance, and three had gametocytes detected. A clear association between low/high *P. falciparum* parasitaemia and duration of positive PCR detections was not found. One patient with late positive PCR was of Norwegian origin, while five were from sub-Saharan Africa and had lived from 10–46 years in Norway. Two of the patients with late positive PCR were immunodeficient with HIV or sickle cell disease.

**Table 2:** PCR results associated with microscopy findings and clinical characteristics

Patient	Species	No. days since treatment	Microscopy (parasite%)	PCR	Fever duration before treatment (days)	Treatment	Origin/years out of endemic area	Comorbidity
No. 1	*P.f* + *P.m*	0	Pos (2)	Pos	35	AL	SSA/1	HIV
		1	Pos (1)	Pos^b,c^				
No. 2	*P.f*	0	Pos (4)	Pos	2	A, AL	SSA/8	HIV
		1	Pos (4)	Pos^b,c^				
No. 3	*P.f*	0	Pos (12)		3	A, AP	Norway	
		1	Neg	Pos				
		2	Neg	Pos				
		11	Neg	Pos^b^				
No. 4	*P.f*	0	Pos (< 1)^a^		38	AP	SSA/10	
		31	Neg	Neg				
No. 5	*P.f*	0	Pos (< 1)		7	AL	SSA/46	
		32	Neg	Pos				
No. 6	*P.f*	0	Pos (2)	Pos	9	Q, A, AL	SSA/21	
		1	Pos (< 0.5)	Pos^b,c^				
		37	Neg	Pos				
No. 7	*P.f*	0	Pos (10)	Pos	21	A, AL	SSA/10	HIV
		4	Neg	Pos				
		11	Neg	Pos				
		39	Neg	Neg				
No. 8	*P.f*	0	Pos (30)	Pos	3	A,	Norway	
		1	Pos (6)	Pos^b,c^		Erythrocyte		
		45	Neg	Pos		apheresis, AL		
No. 9	*P.f*	0	Pos (6)^a^		13	A, AP, AL	SSA/17	Sickle cell disease
		49	Neg	Pos				
No.10	*P.v*	0	Pos (0.5)		10	AP, P	SEA/30	
		2	Pos^a^	Pos^b,c^				
No. 11	*P.v*	0	Pos (0.5)^a^		Not known	AP, P	SSA	
		6	Neg	Pos				
No. 12	*P.v*	0	Pos	Pos	2	A, AP, P	Norway	
		23	Neg	Neg				
No. 13	*P.v*	0	Pos		6	C, P	Norway	
		54	Neg	Neg				

*AL* artemether-lumefantrine, *A* artesunate IV (intravenous), *PA* atovaquone-proguanil, *Q* quinine IV, *C* chloroquine, *P* primaquine, *HIV* human immunodeficiency virus, *SSA* sub-Saharan Africa, *SEA* Southeast Asia

^a^Gametocytes seen by microscopy

^b^Detection of 18S mRNA transcripts (Produced by all parasite stages)

^c^Detection of gametocyte-specific mRNA

## Discussion

In this observational study, DNA-based PCR was positive up to seven weeks after effective malaria treatment. No patients had clinical signs of recrudescence, and there was no risk of re-infection. This is in line with a previous report that found positive malaria PCR after six weeks in a group of returned travellers in Sweden [1]. Positive PCR for weeks after effective treatment of infections is a common phenomenon; a study investigating detectable DNA after treatment of *Chlamydia trachomatis*, *Neisseria gonorrhoeae* and *Trichomonas vaginalis* infections, reported that PCR could be positive up to three weeks post-treatment [15].

Molecular studies have shown that most individuals with asexual parasites also have sub-microscopic gametocyte carriage [16]. In general, the level of circulating gametocytes is low, about 5% compared to other parasite-stages. Gametocytes were detected by microscopy and/or RT-PCR in 67% (8/12) of the patients in the present study. However, gametocyte-specific mRNA was not detected in the late follow-up samples, similar to that reported by Vafa Homann et al*.* in Sweden [1], which might indicate that the detection of *Plasmodium* DNA origin from residuals of destroyed parasites. The phagocytic system has the potential to remove up to 40–80% of malaria infected red blood cells (RBCs) in a few days, but due to sequestration of infected RBCs in organs, the time of complete clearance of parasite residuals is unknown [17]. Drug treatment may also have contributed to clear gametocytes. All the *P. falciparum* patients were treated with artemisinin, which has some gametocidal effect, and all *P. vivax* patients were treated with PQ, which has a strong gametocidal effect. In a study from Kenya and Tanzania estimating gametocyte carriage following treatment with non-artemisinin drugs, artemisinin, and artemisinin in combination with PQ, duration of gametocytaemia was 55, 13 and 6 days, respectively [4].

Collection, handling and analysis of mRNA is challenging [18, 19], and due to the instability of mRNA *versus* DNA, difference in sensitivity between the methods can be a factor that may underestimate late gametocytaemia in this study. A study investigating a similar gametocyte-specific *Pfs*25 RT-PCR, reported that in dilution series down to 0.05 and 0.01 gametocytes/µl, the lowest density samples were often negative [20]. In the present study, the quantitative values detected by *cytb* DNA PCR in samples > 30 days after treatment correspond to ≤ 0.05 gametocytes/µl, so potentially these could have gametocytaemia below detection level of mRNA RT-PCR.

In the study from Kenya and Tanzania investigating gametocytaemia, a nucleic acid sequence based amplification (NASBA) method detecting *Pfs*25 mRNA was applied, a method slightly more sensitive than *Pfs*25 RT-PCR; gametocytes were found in 5–78% of the samples at day 14, and 12–48% at day 28 [4]. However, compared to returned travellers in the present study, patients in malaria-endemic areas may have a higher level of gametocyte carriage post-treatment due to reasons such as late admission and delayed treatment, use of less effective drugs, self-medication with sub-optimal regimes, re-infection, or gametocyte carriage that originate from previous undetected low-density infections.

Looking only at the two DNA-based PCR assays, the *cytb* PCR detected more late follow-up samples than *var*ATS PCR. Hypothetically this could be explained by detection of gametocytes, since the *cytb* method detect mitochondrial DNA present in large amounts in gametocytes. In regard to copy number, the *cytb* PCR is about 2.5 times more sensitive in detecting gametocytes than *var*ATS PCR, while the *var*ATS PCR is about three times more sensitive than *cytb* PCR in detecting asexual parasites. In a study applying field samples from Tanzania, the sensitivity in detecting low level parasitaemia by different real-time malaria PCR methods where compared, and the *var*ATS PCR was then found to be more sensitive than *cytb* PCR [9].

Two samples 11 days after treatment were positive by 18S rRNA RT-PCR (mRNA), but not by the gametocyte specific *Pfs*25 RT-PCR, indicating that possible live asexual parasites circulated in the bloodstream in low densities at this point. Although, negative gametocyte specific *Pfs*25 RT-PCR due to lower sensitivity could also be possible. The quantitative levels by the DNA PCR after 11 days correspond to < 5 parasites/µl. Post-treatment asexual parasites that survive until they are cleared by the immune system continue to produce gametocytes, which also could support the reported phenomenon of gametocyte carriage for several weeks after treatment [4].

For the *P. vivax* samples, a high level of mRNA from gametocytes was detected by the *Pvs*25 RT-PCR at day 2 after treatment, but both mRNA assays were negative for the late follow-up samples. The *cytb* DNA PCR was positive at day 6, though with parasitaemia as low as 0.025 parasites/µl. These results are consistent with the short duration of gametocytaemia in *P. vivax* [2]. The results for *P. vivax* also support that persistent positive *P. falciparum* PCR could be caused by gametocytes; if positive *P. falciparum* PCR was caused only by DNA residuals, it would be expected that also *P. vivax* was PCR positive days/weeks after treatment similar in *P. falciparum* infections. An association between high *P. falciparum* parasitaemia and persistent PCR would intuitively be expected. However, similar to that reported by Vafa Homann et al*.* among Swedish travellers [1], no clear association between low/high level of parasitaemia and persistent positive PCR was identified in the present study.

## Conclusions

DNA-based PCR can be positive up to at least seven weeks after curative malaria treatment, potentially leading to over-diagnose of recrudescence and re-infections. Based on the observations in this study, it is unclear if the DNA origin from residuals of destroyed parasites or live gametocytes. Further studies of both *P. vivax* and *P. falciparum* are needed, since different gametocytaemia biology and treatment-regimes have the potential to give informative answers.

## Data Availability

The datasets used during the current study are available from the corresponding author on reasonable request.

## Abbreviations

PCR: Polymerase chain reaction
PQ: Primaquine (PQ)
*var*ATS: *var* Gene acidic terminal sequence
*cytb*: Cytochrome b
RT: Reverse transcript
AL: Artemether-lumefantrin
AP: Atovaquone-proguanil
RBCs: Red blood cells
